# Do Patients With Acute Coronary Syndrome Face Higher Mortality on Weekends Versus Weekdays? A Comprehensive Analysis of Demographic, Geographic, and Temporal Trends in the United States

**DOI:** 10.1002/clc.70175

**Published:** 2025-07-09

**Authors:** Abdalhakim Shubietah, Abubakar Nazir, Mohamed S. Elgendy, Ameer Awashra, Jehad Zeidalkilani, Mohammad Alqadi, Suleiman Khreshi

**Affiliations:** ^1^ Department of Internal Medicine Advocate Illinois Masonic Medical Center Chicago Illinois USA; ^2^ Oli Health Magazine Organization Kigali Rwanda; ^3^ Faculty of Medicine King Edward Medical University Lahore Pakistan; ^4^ Faculty of Medicine Tanta University Tanta Egypt; ^5^ Department of Medicine An‐Najah National University Nablus West Bank Palestine; ^6^ Department of Internal Medicine MercyOne Siouxland Medical Center Sioux City Iowa USA; ^7^ Department of Internal Medicine University of Toledo Toledo Ohio USA; ^8^ Department of Internal Medicine St. Mary Medical Center, Trinity Health Mid‐Atlantic Langhorne Pennsylvania USA

**Keywords:** acute coronary syndrome, health disparities, racial and ethnic disparities in ACS outcomes, weekday versus weekend, weekend effect

## Abstract

**Background:**

The impact of a “weekend effect” on US acute coronary syndrome (ACS) mortality remains uncertain. We compared weekend and weekday age‐adjusted mortality rates (AAMRs) and analyzed demographic, geographic, and temporal trends from 1999 to 2020.

**Methods:**

We conducted a national analysis of ACS deaths (age ≥ 25 years) using CDC WONDER (ICD‐10: I20.0; I21.0–I21.4; I21.9; I22.0–I22.9; I24.8; I24.9). Crude and AAMRs (per 100 000; 2000 U.S. standard) were calculated, and trends were assessed by joinpoint regression to estimate annual percent changes (APCs) and average APCs (AAPCs).

**Results:**

From 1999 to 2020, there were 3, 101, 451 ACS deaths: 2, 222, 468 on weekdays (AAMR 46.4; 95% CI 46.39–46.51) and 878, 983 on weekends (AAMR 18.4), a 2.5:1 ratio. Both periods saw two‐phase declines—APCs of ≈ –6.4%/year before 2009–2010 and –3.3 to –3.7%/year thereafter (all *p* <  0.001). Disparities persisted: Black adults had the highest AAMRs (20.9 weekend; 53.2 weekday), rural rates exceeded urban (28.7 vs. 15.8; 72.0 vs. 40.2), men exceeded women (23.8 vs. 14.0; 60.2 vs. 35.4), and rates rose steeply with age (weekend 0.3–223.0; weekday 0.7–561.0). After 2009, declines slowed, and weekday deaths in Black adults rose after 2018.

**Conclusions:**

The weekend effect on ACS mortality is minimal, with weekday deaths far outnumbering weekend deaths. Persistent—and sometimes widening—disparities by race, rurality, sex, and age highlight the need for equity‐focused interventions, strengthened rural cardiac care, and targeted prevention.

## 1 Introduction

Acute coronary syndrome (ACS)—encompassing ST‐elevation and non–ST–elevation myocardial infarctions (MI) and unstable angina—remains a major public health burden in the United States. Each year, roughly 1.2 million Americans are hospitalized with ACS. Despite decades of guideline‑driven therapy, ACS continues to exact enormous clinical and economic tolls; the 2024 American Heart Association (AHA) update estimates direct and indirect annual costs at nearly $400 billion [1].

Care delivery for ACS has improved markedly with high‑sensitivity troponin testing, regional primary‑PCI networks, and evidence‑based pharmacotherapy, yet inequality in outcome persists. Women receive reperfusion less often and show higher adjusted mortality than men; Black and Hispanic patients have 35%–45% lower odds of revascularization and worse survival after MI, even in contemporary practice [2]. Rural hospitals likewise report higher mortality, longer treatment delays, and lower use of invasive procedures than urban centers [3]. These disparities reveal that advances in technology and guidelines do not automatically translate into equitable, round‑the‑clock care.

Another system‐level issue gaining fresh attention is the “weekend effect”—the tendency for patients admitted or who pass away on weekends, particularly Saturdays and Sundays, to experience poorer outcomes. Earlier registry studies have found that people admitted with ACS on weekends faced higher in‐hospital mortality rates and longer delays in treatment, such as extended door‐to‐balloon times [4]. Potential causes include reduced weekend staffing, limited availability of specialists, and delays in time‐sensitive interventions. Indeed, early studies found that ACS patients admitted on weekends experienced longer door‐to‐angiography and door‐to‐balloon times and lower rates of immediate PCI compared to weekday admissions [5].

A 2023–2024 multicentre analysis confirmed excess deaths and lower utilization of coronary angiography and PCI for rural weekend MI cases, suggesting that staffing and resource limitations persist outside major centres. Conversely, other large contemporary cohorts demonstrate minimal adjusted differences, fuelling debate over whether the weekend effect still meaningfully exists in the modern era [6].

Our study provides a comprehensive analysis of mortality outcomes for ACS patients in the United States, focusing on demographic, geographic, and temporal trends. It uniquely explores potential differences in mortality between weekends and weekdays, using data from the CDC WONDER database to inform strategies for improving nationwide ACS outcomes.

## 2 Methods

### 2.1 Study Design and Database

This population‐based, national observational cohort study included all ACS deaths among US adults aged ≥ 25 years from 1999 through 2020. Mortality and population data were obtained from the Centers for Disease Control and Prevention's Wide‐ranging Online Data for Epidemiologic Research (CDC‐WONDER) database. ACS deaths were identified by International Classification of Diseases, 10th Revision, Clinical Modification codes for unstable angina (I20.0), acute MI (I21.0–I21.4, I21.9), subsequent MI (I22.0–I22.9), and other acute ischemic heart disease (I24.8, I24.9). Because CDC‐WONDER contains anonymized, publicly available data, the study was exempt from institutional review board approval. All methods adhered to the Declaration of Helsinki and followed the Strengthening the Reporting of Observational Studies in Epidemiology (STROBE) guidelines [7].

### 2.2 Outcome Variables

The primary outcome was day‐of‐week classification of ACS deaths, dichotomized into weekend (Saturday to Sunday) versus weekday (Monday to Friday). We restricted analyses to deaths with ACS recorded as the underlying cause; this could not be performed for subgroups with small counts due to data suppression and rate unreliability.

### 2.3 Data Extraction

From each record, we extracted the following variables, which were used to stratify mortality rates: biological sex (male, female); race/Hispanic origin; 10‐year age group (< 35, 35–44, 45–54, 55–64, 65–74, 75–84, ≥ 85 years); state of residence; and urbanization level per the 2013 NCHS six‐category scheme (large central metro; large fringe metro; medium metro; small metro; micropolitan; noncore).

Because CDC WONDER only provides age data in predefined intervals (e.g., 15–24) and doesn't allow extraction for those aged 18 or older, we restricted our analyses to decendents aged 25 or older.

### 2.4 Statistical Analysis

We calculated crude and age‐adjusted mortality rates (AAMR) per 100 000 population, standardizing to the 2000 US population by direct method across seven 10‐year age strata (< 35, 35–44, 45–54, 55–64, 65–74, 75–84, ≥ 85 years) with 95% confidence intervals. Temporal trends in AAMRs were assessed using the NCI Joinpoint Regression Program (v4.9.0.0). Log‐linear segmented models with up to four joinpoints—selected by the Weighted Bayesian Information Criterion—yielded annual percent changes (APCs) for each segment and an average APC (AAPC) across 1999–2020. Empirical‐quantile 95% confidence intervals were generated from 5001 bootstrap resamples, and statistical significance was defined as two‐sided *p* < 0.05. CDC WONDER suppresses any death‐count cells fewer than 20 deaths.

## 3 Results

### 3.1 Weekday Mortality (Monday to Friday)

From 1999 through 2020, 2, 222, 468 US adults aged ≥ 25 years died of ACS on weekdays, corresponding to an AAMR of 46.4 (95% CI 46.39–46.51). Men experienced a 1.7‐fold higher AAMR than women (60.2 vs. 35.4). Mortality increased steeply with age, rising from a crude rate of 0.66 in persons 25–34 years to 561.0 in those ≥ 85 years. Across race‐ethnicity strata, Black adults had the greatest burden (AAMR 53.2), followed by White (46.7), American Indian/Alaska Native (32.0), and Asian/Pacific Islander adults (23.9); non‐Hispanics exceeded Hispanics (47.5 vs. 33.8). Rural residence amplified risk: AAMR rose from 40.2 in large‐central metropolitan counties to 72.0 in non‐core rural counties. Most weekday deaths occurred in a hospital (36.3%) or at home (24.9%), with smaller proportions in emergency/outpatient settings (18.7%) and long‐term‐care facilities (14.0%). State‐level AAMRs varied more than fourfold, ranging from 23.2 in Alaska to 101.9 in Arkansas.

Joinpoint regression revealed a single inflection in 2010 (95% CI 2009–2011). The AAMR declined by 6.33% per year from 1999 to 2010 (95% CI –6.73 to –6.00; *p* < 0.0001) and by 3.29% per year thereafter (2010–2020; 95% CI –3.74 to –2.73; *p* < 0.0001), yielding an AAPC of –4.90% (95% CI –5.06 to –4.74; *p* < 0.0001). Among women, three joinpoints (2002, 2006, 2011) marked progressively slower annual declines (–4.56%, –7.83%, –5.76%, and –3.86%, respectively; all *p* < 0.0001), resulting in an AAPC of –5.18% (95% CI –5.30 to –5.04). Men showed a single 2010 joinpoint with APCs of –6.31% and –2.96% (*p* < 0.0001), giving an AAPC of –4.73% (95% CI –4.88 to –4.58). Race‐specific patterns differed: American Indian/Alaska Native adults exhibited a biphasic decline (–7.33% then –4.97%) before plateauing after 2018; Asian/Pacific Islander adults showed a 2014 joinpoint with attenuation of the decline thereafter; Black adults displayed joinpoints in 2002, 2010, and 2018 with recent nonsignificant uptick; and White adults paralleled overall trends, each group retaining significant overall declines (all AAPCs –4.48% to –5.07%, *p* < 0.0001).

### 3.2 Weekend Mortality (Saturday to Sunday)

During the same interval, 878, 983 adults died of ACS on weekends, yielding an AAMR of 18.4 deaths. Men accounted for 54.8% of these deaths and had an AAMR of 23.8 versus 14.0 in women. Black adults again had the highest race‐specific AAMR (20.9), followed by White (18.5) and American Indian/Alaska Native (12.6); Asian/Pacific Islander adults had the lowest rate (9.3), and Hispanics experienced lower mortality than non‐Hispanics (13.2 vs. 18.8). Crude mortality escalated with age (0.30 in 25–34 years to 223 in ≥ 85 years) and with increasing rurality (15.8 in large‐central metros to 28.7 in non‐core counties). State‐specific AAMRs ranged sixfold, from 8.7 in Alaska to 40.7 in Arkansas. Inpatient hospital deaths predominated (35.2%), followed by home (24.7%), emergency/outpatient (19.4%), and nursing‐home/long‐term‐care settings (14.6%); fewer than 4% occurred in hospice or other locations.

A single 2009 joinpoint (95% CI 2007–2011) was detected. The AAMR fell by 6.41% per year from 1999 to 2009 (95% CI –7.12 to –5.95; *p* < 0.001) and by 3.65% per year thereafter (95% CI –4.13 to –2.90; *p* = 0.0004), for an overall AAPC of –4.97% (95% CI –5.18 to –4.78; *p* < 0.001). Female mortality declined by 6.46% annually until 2010 and by 4.07% thereafter (AAPC –5.33%; *p* < 0.001), whereas male mortality declined by 6.45% and 3.34% across the same periods (AAPC –4.83%; *p* < 0.001). Among racial–ethnic groups, American Indian/Alaska Native adults experienced a steady reduction (AAPC –5.44%; *p* < 0.001); Asian/Pacific Islander adults had a 2014 joinpoint with subsequent nonsignificant change (AAPC –4.55%; *p* < 0.001); Black adults' 2011 joinpoint yielded APCs of –6.23% and –3.23% (AAPC –4.96%; *p* < 0.001); and White adults mirrored overall trends (APCs –6.33% and –3.58%; AAPC –4.90%; *p* < 0.001).

**Figure 1:**
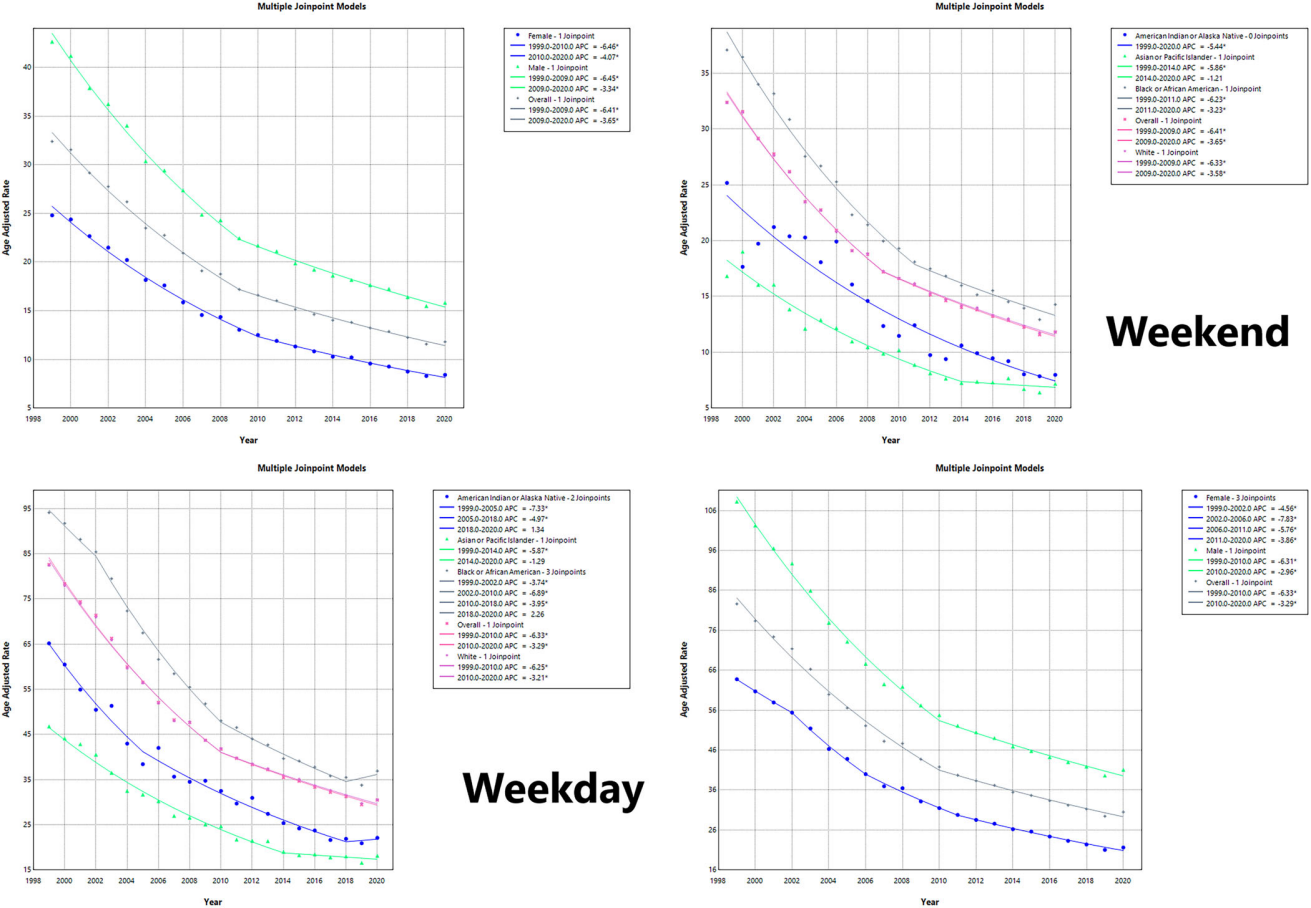
Joinpoint regression of age‐adjusted acute coronary syndrome mortality (deaths/100, 000) in the United States adults ≥ 25 years, 1999–2020: weekend (upper panels) versus weekday (lower panels) trends stratified by sex and race/ethnicity.

**Figure 2:**
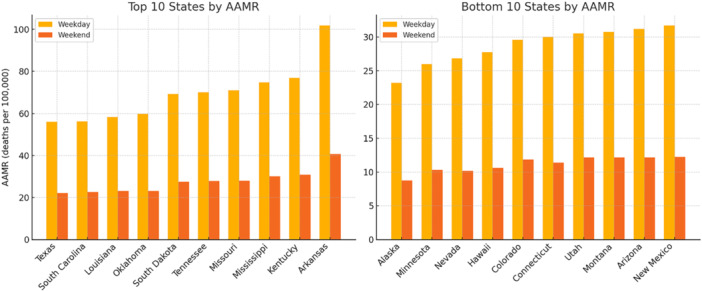
Top and bottom 10 US states by AAMR for weekday versus weekend acute coronary syndrome deaths, 1999–2020.

Baseline demographic characteristics of weekday and weekend ACS decedents are presented in Tables 1 and 2, respectively; joinpoint regression analyses appear in Figure 1; and the top and bottom 10 states by AAMR are shown in Figure 2.

**Table 1:** Baseline characteristics of weekday ACS decedents.

Characteristic	Category	Deaths	Rate
Sex	Female	1, 007, 501	35.4
	Male	1, 214, 967	60.2
Race	Asian or Pacific Islander	42, 551	23.9
	American Indian or Alaska Native	10, 637	32.0
	Black or African American	236, 704	53.2
	White	1, 932, 576	46.7
Hispanic origin	Hispanic or Latino	115, 281	33.8
	Not Hispanic or Latino	2, 101, 843	47.5
Age group (years)^†^	25–34	6059	0.7
	35–44	37, 264	4.0
	45–54	142, 133	15.3
	55–64	300, 139	39.2
	65–74	439, 253	86.1
	75–84	627, 185	210.1
	≥ 85	670, 435	561.0
Urbanization	Large central metro	527, 429	40.2
	Large fringe metro	464, 361	40.7
	Medium metro	441, 257	43.5
	Small metro	237, 356	50.8
	Micropolitan (Nonmetro)	288, 401	61.7
	Noncore (Nonmetro)	263, 664	72.0
Place of death^‡^	Inpatient hospital	807, 490	36.3%
	Home	554, 362	24.9%
	Emergency/outpatient	415, 632	18.7%
	Long‐term‐care facility	310, 866	14.0%

*Note:* Rate per 100 000: age‐adjusted mortality rate (AAMR) except ^†^crude mortality rate and ^‡^percentage of total weekday deaths.

**Table 2:** Baseline characteristics of weekend ACS decedents.

Characteristic	Category	Deaths	Rate
Sex	Female	397, 488	14.0
	Male	481, 495	23.8
Race	Asian or Pacific Islander	16, 658	9.3
	American Indian or Alaska Native	4, 188	12.6
	Black or African American	69, 439	20.9
	White	765, 087	18.5
Hispanic origin	Hispanic or Latino	100, 634	13.2
	Not Hispanic or Latino	777, 567	18.8
Age group (years)^†^	25–34	1, 127	0.3
	35–44	8, 654	1.6
	45–54	25, 389	5.0
	55–64	74, 523	15.1
	65–74	161, 487	41.0
	75–84	246, 588	89.5
	≥ 85	269, 215	223.0
Urbanization	Large central metro	297, 456	15.8
	Large fringe metro	264, 721	16.1
	Medium metro	163, 042	17.2
	Small metro	78, 954	19.5
	Micropolitan (Nonmetro)	33, 061	21.9
	Noncore (Nonmetro)	41, 749	28.7
Place of death^‡^	Inpatient hospital	309, 077	35.2%
	Home	217, 089	24.7%
	Emergency/outpatient	170, 743	19.4%
	Long‐term‐care facility	128, 087	14.6%
	Other (hospice, unknown)	53, 487	6.1%

*Note:* Rate per 100 000: age‐adjusted mortality rate (AAMR) except ^†^crude mortality rate and ^‡^percentage of total weekday deaths.

## 4 Discussion

This comprehensive national analysis spanning 1999–2020, a 22‐year nationwide analysis, reveals significant and nuanced disparities in ACS mortality across temporal (weekend vs. weekday), demographic, geographic, and racial/ethnic strata. Using CDC WONDER data, we identified over 3.1 million ACS deaths, with distinct patterns in AAMRs and longitudinal trends that underscore persistent gaps in cardiovascular care and outcomes across the United States.

### 4.1 Temporal Trends and Recent Upticks

Over the 22‐year period, both weekend and weekday ACS mortality showed statistically significant two‐phase declines. The decline in ACS mortality across the US was marked by a pronounced inflection point in 2009, after which the rate of improvement slowed considerably. Before 2009, weekend ACS deaths declined at an APC of –6.41%, followed by a slower APC of –3.65% thereafter, resulting in a net AAPC of –4.97%. These patterns were mirrored in weekday trends. Weekday APCs showed a similar trend (–6.33%, then –3.29%). These declines reflect national progress in cardiovascular care, including widespread adoption of reperfusion therapy, secondary prevention, and systems‐based STEMI management [8, 9]. However, our data revealed a concerning post‐2018 uptick in weekday mortality among Black adults, suggesting a reversal of prior gains. This trend may signal widening racial inequities, potentially exacerbated by healthcare access issues, systemic racism, or the early impacts of the COVID‐19 pandemic, which disproportionately affected communities of color and strained cardiovascular care delivery [10].

### 4.2 Weekend Versus Weekday Mortality Patterns

A total of 878, 983 ACS deaths occurred on weekends, corresponding to an AAMR of 18.4, compared to 2, 222, 468 deaths and a substantially higher AAMR of 46.4 on weekdays. The weekday‐to‐weekend mortality rate ratio was approximately 2.5:1, suggesting that although fewer events occur on weekends, the burden remains clinically and epidemiologically significant.

Sex disparities were consistent across both periods, with men exhibiting markedly higher AAMRs than women (weekend: 23.8 vs. 14.0; weekday: 60.2 vs. 35.4), aligning with long‐established sex‐based differences in cardiovascular disease prevalence and outcomes. This male excess is consistent with long‐standing sex‐based differences in the epidemiology and prognosis of cardiovascular disease and probably reflects both biological vulnerability and differences in health‐care‐seeking behaviour. Our findings reinforce that men continue to bear a higher global burden of ischemic heart disease, largely driven by greater exposure to behavioral risk factors like smoking and physical inactivity [11, 12]. Although the age‐standardized mortality rate and age‐standardized prevalence rate have declined in both sexes, the rate of decline is slower in men [12]. Women, in turn, face unique sex‐specific hazards—including hormonal fluctuations and pregnancy‐related conditions—that sustain or widen disparities in risk‐factor exposure. These trends underscore persistent and evolving sex‐based differences in cardiovascular risk and outcomes [11, 12].

### 4.3 Racial and Ethnic Disparities

Marked disparities in ACS mortality by race and ethnicity persist, despite overall declines. Clear racial disparities emerged, with Black adults experiencing the highest AAMRs of 20.9 on weekends and 53.2 on weekdays, well above national averages. These findings reinforce CDC data highlighting the disproportionate burden of cardiovascular disease among Black populations, potentially driven by a confluence of structural inequities, comorbid conditions, and barriers to timely care [13]. According to the AHA, about 60% of Black adults in the United States aged 20 and older have some form of cardiovascular disease such as coronary heart disease, heart failure, stroke, or high blood pressure—compared to approximately 49% of all US adults in the same age group [13, 14]. Joinpoint regression revealed distinct temporal dynamics: Black adults experienced a turning point in 2011 with a post‐2011 APC of –3.23%, slower than earlier reductions (–6.23%) and suggesting stagnation in gains. In contrast, Asian/Pacific Islanders had the lowest AAMRs (9.3 on weekends, 23.9 on weekdays), consistent with prior reports suggesting a comparatively lower incidence of ischemic cardiovascular events in this group. Notably, Asian/Pacific Islanders saw an even more pronounced plateau after 2014, with a non‐significant decline (–1.21%) thereafter, raising concerns about access or cultural barriers in these communities.

Furthermore, the “steady decline” in American Indian/Alaska Natives (AAPC –5.44%) masks broader health challenges these populations face, especially considering access constraints in rural and reservation‐based healthcare systems. Hispanic individuals exhibited lower mortality rates than non‐Hispanics across both temporal categories (weekend: 13.2 vs. 18.8; weekday: 33.8 vs. 47.5), which may be influenced by age distribution and protective cultural or behavioral factors often referred to as the “Hispanic paradox.” [15].

### 4.4 Sex‐Specific Analyses

Sex‐specific analyses revealed slightly steeper early declines in females (–6.46% vs. –6.45% in males), but a more sustained late‐phase decrease in women (–4.07% vs. –3.34%), yielding a slightly greater overall AAPC (–5.33% vs. –4.83%). These patterns suggest improvements in female‐specific ACS recognition and treatment, although the consistently lower AAMRs in women (14.0 vs. 23.8) also reflect biological and behavioral factors, including delayed symptom onset and differences in presentation [12].

### 4.5 Age‐Specific Trends

ACS mortality increased steeply with age across both periods. Crude ACS mortality rose steeply with age. Weekend AAMRs rose from 0.30 in those aged 25–34 to 223.0 in individuals ≥ 85 years, while weekday rates escalated from 0.66 to 561.0 in the same age brackets. The exponential age gradient highlights the compounded risk of ACS with advancing age and underscores the importance of targeted prevention strategies in older adults, particularly given aging population trends [16].

### 4.6 Geographic Variation and Urban‐Rural Disparities

Geographic variability was substantial. State‐level AAMRs on weekends ranged sixfold, from 8.7 to 40.7, and on weekdays, nearly fourfold, from 23.2 to 101.9, reflecting regional disparities in population health, healthcare infrastructure, and emergency response systems. Rural residents experienced markedly higher AAMRs than urban residents (weekend: 28.7 vs. 15.8; weekday: 72.0 vs. 40.2). These inequities likely arise from delayed presentation, limited access to advanced cardiac care centers, and under‐resourced health systems in rural communities. Such findings underscore complex rural–urban gaps in cardiovascular outcomes, stemming from differences in demographic composition, proximity to medical services, and overall quality of care [17, 18]. The data strongly align with CDC initiatives to reduce rural health disparities and emphasize the need for expanding tele‐cardiology, EMS capabilities, and 24/7 STEMI networks in underserved areas [18].

### 4.7 Place of Death and Implications for Emergency Response

Place‐of‐death patterns reveal further insight into systemic gaps. Most ACS deaths occurred in inpatient hospitals (35.2%), but nearly 45% occurred outside of hospitals, mainly at home (24.7%), in emergency/outpatient settings (19.4%), or in long‐term care facilities (14.6%). Less than 4% occurred in hospice or other managed‐care environments. These findings support existing evidence and highlight potential delays in presentation, diagnostic uncertainty in early symptoms, and underutilization of palliative or advanced care planning in end‐stage cardiac disease [19, 20, 21].

### 4.8 Implications for Practice and Policy

Our findings hold important implications for public health policy, health equity, and acute care systems. The persistent disparities in ACS mortality, particularly by race, geography, and age, mirror broader challenges identified in CDC reports and call for targeted interventions. Strategies should include expansion of cardiovascular screening and risk management programs in high‐burden populations, strengthening rural healthcare capacity and transport systems, reinforcing public education on ACS symptom recognition and timely care‐seeking behavior, and addressing structural determinants of health through policy reform and community engagement.

### 4.9 Limitations

This study is subject to several important limitations. First, death‐certificate coding is imperfect. Misclassification of the underlying cause of death—especially for out‐of‐hospital events with limited diagnostic confirmation—may bias rates despite our use of a comprehensive list of International Classification of Diseases, 10th Revision codes for ACSs. Shifts in coding practice and the 2010 introduction of high‐sensitivity troponin assays could also alter the likelihood that ACS is recorded, independent of true incidence. Second, CDC WONDER provides only aggregate mortality data. The absence of individual‐level information on comorbidities, medications, symptom onset, treatment times, socioeconomic status, and insurance precludes adjustment for key confounders and limits causal inference; associations observed at the population level may not hold for individual patients (ecological fallacy). Third, several sources of measurement error remain. Population denominators rely on intercensal projections that may be less accurate for small or rural jurisdictions; counts < 10 are suppressed and rates based on < 20 deaths flagged as unreliable, introducing imprecision in subgroup analyses. Day‐of‐week assignment is based on recorded date of death rather than symptom onset, so events that cross midnight could blur weekday–weekend boundaries. Residence is recorded as usual home address, not location of the fatal event, potentially biasing state‐level estimates for border counties. Fourth, the analysis captures only fatal events. Non‐fatal MI, upstream preventive care, and regional differences in emergency cardiac capacity are not represented, yet they likely influence the demographic and geographic mortality gradients observed. Collectively, these limitations mean that our findings should be interpreted as population‐level associations rather than definitive evidence of cause‐effect relationships.

## 5 Conclusion

This 22‐year, nationwide analysis demonstrates that US adults with ACS do not experience higher age‐adjusted mortality on weekends than on weekdays; instead, weekday deaths predominate both in absolute numbers (≈2.2 million vs. 0.88 million) and in rates (46.4 vs. 18.4). The “weekend effect,” therefore, appears largely attenuated at the population level. Far more consequential are the enduring—and in some subgroups widening—disparities by race, rurality, sex, and age. Black Americans, residents of the most remote rural counties, and adults aged ≥ 85 years bear the heaviest toll.

These findings underscore three priorities. First, equity‐focused quality improvement is essential: expanding 24/7 STEMI networks, tele‐cardiology, and early‐warning public education can narrow rural and racial gaps that persist despite overall advances. Second, targeted prevention and rapid‐response pathways for high‐risk groups, particularly older adults and Black communities, are needed to sustain momentum in mortality reduction. Third, richer, patient‐level data are imperative to disentangle the contributions of comorbidities, socioeconomic status, and care delays that could not be captured in death‐certificate records.

## Author Contributions

Abdalhakim Shubietah, Abubakar Nazir, and Mohamed S. Elgendy wrote the original draft and supervised the work. Abdalhakim Shubietah downloaded and analyzed the data. Jehad Zeidalkilani and Suliman Khreshi provided additional supervision. Mohammad Alqadi conducted the literature search. Ameer Awashra critically revised the draft. All authors reviewed and approved the final manuscript.

## Ethics Statement

This study used de‐identified, publicly available mortality data from the CDC WONDER database and was therefore exempt from institutional review board oversight.

## Consent

The authors have nothing to report.

## Conflicts of Interest

The authors declare no conflicts of interest.

## Data Availability

All data are publicly available from the Centers for Disease Control and Prevention's WONDER database (https://wonder.cdc.gov).

## References

[1] S. S. Martin , A. W. Aday , Z. I. Almarzooq , et al., “2024 Heart Disease and Stroke Statistics: A Report of US and Global Data From the American Heart Association,” Circulation 149, no. 8 (February 2024): e347–e913. 38264914 10.1161/CIR.0000000000001209 PMC12146881

[2] L. G. Glance , K. E. Joynt Maddox , J. Shang , et al., “The COVID‐19 Pandemic and Associated Inequities in Acute Myocardial Infarction Treatment and Outcomes,” JAMA Network Open 6, no. 8 (August 2023): e2330327. 37624599 10.1001/jamanetworkopen.2023.30327 PMC10457721

[3] S. Sritharan , B. Wilsmore , J. Wiggers , et al., “Rural‐Urban Differences in Outcomes of Acute Cardiac Admissions in a Large Health Service,” JACC: Advances 3, no. 11 (November 2024): 101328. 39469611 10.1016/j.jacadv.2024.101328 PMC11513678

[4] M. Khoshchehreh , E. M. Groves , D. Tehrani , A. Amin , P. M. Patel , and S. Malik , “Changes in Mortality on Weekend Versus Weekday Admissions for Acute Coronary Syndrome in the United States Over the Past Decade,” International Journal of Cardiology 210 (May 2016): 164–172. 26950171 10.1016/j.ijcard.2016.02.087 PMC4801736

[5] S. Vallabhajosyula , S. H. Patlolla , P. E. Miller , et al., “Weekend Effect in the Management and Outcomes of Acute Myocardial Infarction in the United States, 2000−2016,” Mayo Clinic Proceedings: Innovations, Quality & Outcomes 4, no. 4 (August 2020): 362–372. 10.1016/j.mayocpiqo.2020.02.004 PMC7411160 32793864

[6] M. F. A. Baig , “Analysis of the Weekend Effect on Mortality, Diagnostic Coronary Angiography, and Percutaneous Coronary Intervention in Acute Myocardial Infarction Across Rural US Hospitals,” Cureus [Internet] 16 (February 2024): e53751, https://www.cureus.com/articles/228191-analysis-of-the-weekend-effect-on-mortality-diagnostic-coronary-angiography-and-percutaneous-coronary-intervention-in-acute-myocardial-infarction-across-rural-us-hospitals. 38465191 10.7759/cureus.53751 PMC10921120

[7] A. A. Ghaferi , T. A. Schwartz , and T. M. Pawlik , “STROBE Reporting Guidelines for Observational Studies,” JAMA Surgery 156, no. 6 (June 2021): 577. 33825815 10.1001/jamasurg.2021.0528

[8] R. K. Wadhera and K. E. Joynt Maddox , “Policy Strategies to Advance Cardiovascular Health in the United States—Building on a Century of Progress,” Circulation. Cardiovascular Quality and Outcomes 17, no. 4 (April 2024): 010149, https://www.ahajournals.org/doi/10.1161/CIRCOUTCOMES.123.010149. 10.1161/CIRCOUTCOMES.123.010149 38626057

[9] J. D. Schwalm , P. Joseph , D. Leong , et al., “Cardiovascular Disease In the Americas: Optimizing Primary and Secondary Prevention of Cardiovascular Disease,” Lancet Reg Health—Am 42 (February 2025): 100964. 40034111 10.1016/j.lana.2024.100964 PMC11873640

[10] A. S. Arun , C. Caraballo , M. Sawano , et al., “Cause‐Specific Mortality Rates Among the US Black Population,” JAMA Network Open 7, no. 9 (September 2024): e2436402. 39348122 10.1001/jamanetworkopen.2024.36402 PMC11443349

[11] M. K. Mahowald , K. Esmail , F. M. Ezzeddine , C. Choi , H. Mieszczanska , and G. Velarde , “Sex Disparities in Cardiovascular Disease,” Methodist DeBakey Cardiovascular Journal 20, no. 2 (March 2024): 107–119. 38495656 10.14797/mdcvj.1328 PMC10941692

[12] M. Mahalleh , R. Narimani‐Javid , K. Izadpanahi , et al., “Hearts Apart: Exploring Sex Disparity in the Global and Regional Burden of Ischemic Heart Disease; A Systematic Analysis From the Global Burden of Disease Study 1990–2021,” BMC Cardiovascular Disorders 25, no. 1 (May 2025): 346. 40316911 10.1186/s12872-025-04770-0 PMC12046674

[13] Cardiovascular Health Risks Continue to Grow within Black Communities , Action Needed | American Heart Association [Internet]. [Accessed May 9, 2025], https://newsroom.heart.org/news/cardiovascular-health-risks-continue-to-grow-within-black-communities-action-needed.

[14] B. Bozkurt , T. Ahmad , K. M. Alexander , et al., “Heart Failure Epidemiology and Outcomes Statistics: A Report of the Heart Failure Society of America,” Journal of Cardiac Failure 29, no. 10 (October 2023): 1412–1451. 37797885 10.1016/j.cardfail.2023.07.006 PMC10864030

[15] J. M. Ruiz , P. Steffen , and T. B. Smith , “Hispanic Mortality Paradox: A Systematic Review and Meta‐Analysis of the Longitudinal Literature,” American Journal of Public Health 103, no. 3 (March 2013): e52–e60. 10.2105/AJPH.2012.301103 PMC3673509 23327278

[16] A. A. Damluji , D. E. Forman , T. Y. Wang , et al., “Management of Acute Coronary Syndrome in the Older Adult Population: A Scientific Statement From the American Heart Association,” Circulation [Internet] 147, no. 3 (January 2023): e32–e62, https://www.ahajournals.org/doi/10.1161/CIR.0000000000001112. 36503287 10.1161/CIR.0000000000001112 PMC10312228

[17] I. Shahid , A. Shoaib , S. Khan , et al., “Urban‐Rural Differences in Atherosclerotic Cardiovascular Disease‐Related Mortality in the United States, 1999−2020,” Journal of the American College of Cardiology 83, no. 13 (April 2024): 1189.

[18] S. H. Cross , M. R. Mehra , D. L. Bhatt , et al., “Rural‐Urban Differences in Cardiovascular Mortality in the US, 1999−2017,” Journal of the American Medical Association 323, no. 18 (May 2020): 1852. 32396176 10.1001/jama.2020.2047 PMC7218488

[19] V. L. Roger , A. S. Go , D. M. Lloyd‐Jones , E. J. Benjamin , et al., “Heart Disease and Stroke Statistics—2012 Update: A Report From the American Heart Association,” Circulation [Internet] 125, no. 1 (2012): e2–e220, https://www.ahajournals.org/doi/10.1161/CIR.0b013e31823ac046. 22179539 10.1161/CIR.0b013e31823ac046 PMC4440543

[20] B. Bozkurt , T. Ahmad , K. Alexander , et al., “HF STATS 2024: Heart Failure Epidemiology and Outcomes Statistics an Updated 2024 Report From the Heart Failure Society of America,” Journal of Cardiac Failure 31, no. 1 (January 2025): 66–116. 39322534 10.1016/j.cardfail.2024.07.001

[21] A. Timmis , P. Vardas , N. Townsend , et al., ”European Society of Cardiology: Cardiovascular Disease Statistics 2021,” European Heart Journal 43, no. 8 (February 2022): 716–799. 35016208 10.1093/eurheartj/ehab892

